# Socioeconomic Status and Clinical Outcomes in Chronic Kidney Disease: Bootstrap Validation of a Simple Indicator

**DOI:** 10.3390/jcm13123600

**Published:** 2024-06-20

**Authors:** Annalisa Pitino, Graziella D’Arrigo, Carmela Marino, Patrizia Pizzini, Graziella Caridi, Francesca Mallamaci, Giovanni Tripepi, Carmine Zoccali

**Affiliations:** 1 National Research Council of ITALY (CNR), Institute of Clinical Physiology in Rome, 00042 Rome, Italy; 2 National Research Council of ITALY (CNR), Institute of Clinical Physiology in Reggio Calabria, 00185 Reggio Calabria, Italy; 3 Nephrology and Renal Transplantation Unit, Grande Ospedale Metropolitano, 20126 Reggio Calabria, Italy; 4 Renal Research Institute, New York, NY 10065, USA; 5 Institute of Molecular Biology and Genetics (Biogem), 21846 Ariano Irpino, Italy; 6 Carmine Zoccali IPNET, Associazione Ipertensione Nefrologia Trapianto (IPNET), c/o Nefrologia, Grande Ospedale Metropolitano, 89124 Reggio Calabria, Italy

**Keywords:** socioeconomic status, quality of life, CKD

## Abstract

**Background:** Chronic Kidney Disease (CKD) is a complex health condition that interacts significantly with socioeconomic determinants, particularly income status and education. This study developed a simple indicator of socioeconomic status (SES), which is composed of income status and education in CKD patients, and evaluated its impact on health outcomes in this population. **Methods:** This study was conducted on 561 CKD patients, stages 2–5. The composite SES score was developed by combining the regression coefficients of income and education as predictors of the study endpoint in a multivariable Cox model, normalizing these coefficients to derive weights, and then using these weights to calculate an individual percentage score based on each person’s income and education. The composed SES indicator was internally validated through bootstrap analysis. Over a median follow-up time of 36 months, we tracked all-cause death and non-fatal cardiovascular events. **Results:** Both lack of income (*p* = 0.020) and low educational level (*p* = 0.034) were independently related to the combined endpoint. Based on these covariates‘ regression coefficients, a composite socioeconomic score considering income and educational level was generated. In a Cox regression model, a 10% increase in this composite risk score entailed a 25% increase in the hazard ratio (HR) of the combined endpoint [HR (10% increase): 1.25], and the internally validated 95% CI ranged from 1.14 to 1.41 (*p* < 0.001). **Conclusions:** This study underscores the significant impact of a simple, bootstrap-validated composite SES indicator on CKD patients’ health outcomes. These findings highlight the importance of considering education and socioeconomic factors in managing and treating CKD patients and inform future research and policy considerations for this population.

## 1. Introduction

Chronic Kidney Disease (CKD) is a common, multifaceted condition affecting over 800,000 persons worldwide [1]. It has been proven that socioeconomic status (SES) is an important factor influencing the outcome of CKD [cite reference]. Individuals with lower SES are more likely to experience adverse outcomes, such as cardiovascular disease [2], higher rates of mental health disorders [3], a higher prevalence of chronic diseases such as diabetes and chronic obstructive pulmonary disease (COPD) [4], higher overall mortality rates [5], and kidney failure [6]. Access to health information, race and ethnicity, and quality healthcare services may contribute to this disparity [7].

Many indicators are widely used in evaluating SES in CKD populations. Income is a significant correlate of symptom burden in kidney failure patients [8]. A higher education level is associated with better outcomes in CKD patients [9]. However, there are also limitations when using education level as an indicator of SES [10]. Other indicators, like occupation, wealth, and geographic location, can also partially capture the SES of CKD populations [11]. However, the lack of a unified definition of SES may lead to an underestimated influence of the impact from SES on CKD [12]. Hence, an indicator based on easily obtained information that comprehensively demonstrates patients’ SES is needed.

Income level is a robust indicator of SES [12]. Combining income and education levels can offer a more holistic view of patients’ socioeconomic standing [13]. By using a composite indicator based on income and education levels, researchers and policymakers can better understand the SES of patients and the influence of SES on health outcomes.

In this study, we used Cox regression analyses to create an indicator based on income and education levels to better represent the SES of CKD patients, and further evaluate the predictive ability of this indicator by exploring how it is associated with adverse outcomes such as mortality and cardiovascular events in CKD patients by using bootstrap analysis.

## 2. Materials and Methods

The study protocol was approved by ethical committees of our institutions Riuniti Hospital, Calabria Region, Italy (Approval code n. 35 of 11 November 2004) and informed consent was obtained from each participant. Data collection was conducted ensuring the privacy and confidentiality of all participant information. All study procedures were conducted in accordance with the Declaration of Helsinki and other relevant ethical guidelines.

### 2.1. Patients

The source population was composed of 759 stage 2–5 CKD patients consecutively recruited from nephrology units in southern Italy (Calabria, Sardinia, and Sicily regions) participating in the Multiple Intervention and Audit in Renal Diseases to Optimize Care (MAURO) study (Table 1). The MAURO study was a cluster randomized controlled trial in 22 renal clinics that aimed to assess the efficacy of a multimodal quality improvement intervention to increase compliance with guideline recommendations for preventing CKD progression and CV complications in a CKD population. A total of 198 patients out of 759 were excluded because of missing information about income. Thus, in the present analysis, we included 561 patients (74%) with stage 2–5 CKD (age 61 ± 11 years; 61% male). Included patients had a lower prevalence of diabetics, systolic blood pressure, and CRP and higher hemoglobin as compared to those excluded because of missing data (Supplementary Table S1). Patient enrolment was performed between October 2005 and 2008. The selection criteria and the detailed clinical characteristics of the MAURO cohort are described elsewhere [14,15]. Briefly, inclusion criteria were as follows: nonacute or rapidly evolving renal diseases, age ranging from 18 to 75 years, non-transplanted, nonpregnant, and not affected by cancer or diseases in the terminal phase. This study included six visits over a 3-year follow-up. All patients were in stable clinical condition at enrolment and none had intercurrent infections or acute inflammatory processes.

### 2.2. Follow-Up and Study Outcome

After the initial assessment, patients were followed up for a median time of 36 months (interquartile range 35–37 months). All-cause death and non-fatal cardiovascular (CV) events were accurately recorded across time. These events included myocardial infarction, documented by electrocardiography and biomarkers of myocardial injury; heart failure, defined as dyspnea in addition to two of the following conditions: raised jugular pressure, bi-basilar crackles, pulmonary venous hypertension, or interstitial edema on chest radiography requiring hospitalization; electrocardiography-documented arrhythmia; stroke; peripheral vascular disease; and major arterial or venous thrombotic episodes. A composite endpoint, “all-cause mortality and non-fatal cardiovascular (CV) events”, was considered for this analysis.

### 2.3. Laboratory Measurements

In the MAURO study, various blood biomarkers were measured in clinical laboratories that routinely verify the accuracy of their testing methods. The above-mentioned markers included total cholesterol, phosphate, and hemoglobin. Serum CRP (C-reactive protein) levels were measured using a high-sensitivity RIA kit, while serum creatinine levels were analyzed using the Jaffe method on multichannel analyzers. Their glomerular filtration rate (GFR) was estimated using the MDRD equation [16].

### 2.4. Office Blood Pressure (BP) Measurements

Office BP was calculated as the average of 2 or 3 measurements at 1 to 2 min intervals during the morning hours. BP measurements were conducted in a sitting position by the attending physician or a nurse with the cuff at heart level using sphygmomanometers, periodically tested and appropriately calibrated.

### 2.5. Quality of Life, Income, and Educational Level

Quality of life was measured by the Kidney Disease Quality of life (KDQOL) short form (KDQOL-SF^TM^), an instrument which measures eight domains of QoL [physical functioning, role physical health, energy fatigue, pain, role emotional problem, emotional well-being, social function, and general health and two summary scores (the physical component score, PCS; and the mental component score, MCS)], which are calculated by a well-validated algorithm [17]. The KDQOL-SF^TM^ measured quality of life in the version translated into Italian and specifically validated in a sample of Italian patients with CKD [18,19].

Information on homeownership, income, educational level, and number of cars per capita was derived from patient interviews administered to all participants at baseline and across longitudinal visits (see Supplementary Table S2). Patients were categorized into three groups: (1) no declared income (the worst condition), (2) declared income from retirement (the intermediate condition), and (3) declared income from work or work plus retirement (the best condition). Educational level was categorized into two levels: equal or lower than primary school and from middle school onward. The assessment of income was performed at baseline and at 1, 2, and 3 years of follow-up.

### 2.6. Statistical Analysis

According to the data distribution and normality evaluation, data are summarized as mean and standard deviation (SD), median and interquartile range (IQR), or absolute frequency and percentage (for categorical variables). Kruskal–Wallis and chi-square tests were used to compare groups (see Table 1), as appropriate.

The independent relationship between income status and educational level and the combined endpoint were investigated by univariable (crude) and multivariable Cox regression analyses. As potential confounders, we considered all variables listed in Table 1. Among these, variables which were associated with both the key exposure (income status) and the outcome (composite endpoint) with *p* ≤ 0.10 were considered in multivariable, region-stratified, models of various complexity. In these models, we did not include the number of cars per capita as well as physical and mental component scores, to avoid over-adjustment, as these variables strongly related to the income groups (Table 1). Including these variables into the Cox models could obscure the true relationship between income sources and mortality by adjusting for factors that are direct consequences of income status. In Cox models, data are expressed as hazard ratio (HR), 95% confidence interval, and *p* value.

Because a lower educational level and a low income are both associated with worse survival, we generated a composite SES risk score based on both income and educational level and adjusted for the full set of variables listed in Table 2, Model 3. To generate the composite SES score, we applied the following procedure: (1) the regression coefficients of income and educational level (i.e., the natural logarithm of the corresponding hazard ratios reported in Table 2, Model 2) were summed up, and (2) the same regression coefficients were then divided by their sum and multiplied by 100, thus deriving a weight for each variable ranging from 0 to a given percentage, with all the remaining covariates set to the corresponding mean value. These weights were then used to generate an individual percentage score based on the individual income and education. The composite SES score was internally validated using a bootstrapping resampling technique [20]. Bootstrapping is a resampling procedure that uses data from one sample to generate a sampling distribution by repeatedly taking random samples from the known sample, with replacement. The data analysis was performed using the statistical software SPSS for Windows Version 28.0.1.1, IBM Corp. (2021), Armonk, NY, USA.

## 3. Results

The present analysis includes 561 stage 2–5 CKD patients (age: 61 ± 11 years, males: 61%). Overall, 112 patients declared no income (20%), 293 patients declared income from retirement (52%), and the remaining 156 patients (28%) declared income from work or work plus retirement (see Table 1). The three groups significantly differed for all variables reported in Table 1 except for total cholesterol, eGFR, and homeownership that were similar among the three groups. In particular, patients who declared no income were those with the lowest MCS value and percentage of males (Table 1). The educational level and the average number of cars per capita were higher in patients who declared income from work or work plus retirement (Table 1) than in the remaining patients. Patients with low income were those with worse values of hemoglobin, and serum phosphate (Table 1). PCS was lower in patients who declared income from retirement than in the remaining two categories (Table 1). As expected, age was highest in patients who declared income from retirement (Table 1). Of note, in 85% of patients, the income status remained stable over time.

### Survival Analysis

During the follow-up period (median 36 months, interquartile range 35–37 months), 93 patients experienced the combined endpoint all-cause death/non-fatal cardiovascular (CV) events (63 patients had non-fatal CV events, 20 patients died for CV causes, and the remaining 10 patients died for causes other than CV). Upon univariable Cox regression analyses, patients who declared income from retirement and those with no income were those with the highest hazard ratios for the combined endpoint as compared to those with income from work or work and retirement (the reference category) (Table 2, crude model). Upon univariable Cox regression analysis, homeownership was largely unrelated to survival (*p* = 0.774) and this was true also when forced into Model 3 in Table 2 (*p* = 0.592). Data adjustment for age [and other risk factors such as male sex, educational level, smoking, and alcohol use] showed that patients with no income were those with the highest risk of the combined endpoint, whereas the intermediate category (i.e., patients with income from retirement) lost prediction power after data adjustment (Table 2, Model 1).

**Figure 1:**
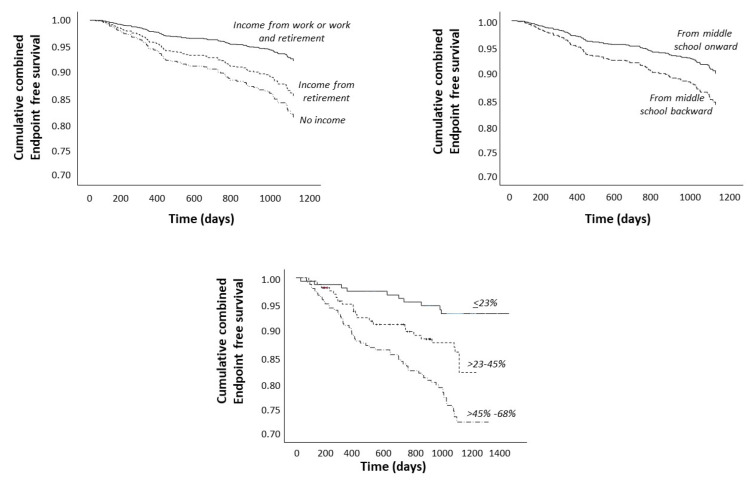
Survival curves by income level (top-left), educational level (top-right), and the combined socioeconomic score (the combination of these variables, bottom). Data were adjusted for variables listed in Table 2, Model 3.

Further data adjustment for diabetes (Table 2, Model 2), hemoglobin, and systolic blood pressure and phosphate (Table 2, Model 3) did not impact these results. As shown graphically in Figure 1, patients with no income, with a relatively low educational level (from middle school backward), and with a socioeconomic score >45–68% were those with the highest risk of the combined endpoint. Patients with a lower educational level had a higher hazard ratio of the combined endpoint, which was 67% higher than that of the remaining patients (HR: 1.67, 95%: 1.04–2.95), independently of income and other potential confounders (Table 2, Model 3). The composite risk score based on income and educational level (see Methods—Statistical Analysis and Table 3) adjusted for variables included in Table 2, Model 3 was 0 in 118 cases, 23% in 38 cases, 32% in 112 cases, 45% in 50 cases, 55% in 181 cases, and 68% in the remaining 62 cases. In a Cox regression model, a 10% increase in the composite risk score entailed a 25% increase in the hazard ratio of the combined endpoint [HR (10% increase): 1.25], and the bootstrap-validated 95% CI was between 1.14 and 1.41, (*p* < 0.001).

## 4. Discussion

In this study, we found that a simple indicator of socioeconomic status based on income data and education level predicted the risk for a relevant combined endpoint, including all-cause death/non-fatal cardiovascular events, in CKD patients. We internally validated our findings, and our data appear to be a promising, simple approach to assess the socioeconomic impact on clinical outcomes in a high-risk population like CKD patients. When externally validated, this simple indicator can be useful in observational studies of the CKD population.

CKD is a prevalent health issue that affects a significant portion of the global population [1], and this condition has a very high risk for death and cardiovascular disease [21]. Numerous factors impact the high risk of CKD [11]. In this regard, the interplay between socioeconomic status (SES) and health outcomes in the context of CKD has been an area of research interest for years. Several studies have highlighted the impact of SES factors such as income, education, occupation, and housing on health outcomes [11].

Our study focuses on a simple indicator based on income status and education level as key CKD management and progression determinants. In the composite SES score computation, we excluded homeownership due to its high prevalence among the study population as well as because it was largely unrelated to the composite endpoint including mortality and cardiovascular events. The relationship between homeownership and health outcomes is complex and multifaceted. A study by Pollack et al. [22] suggests that homeownership can be a double-edged sword, with potential benefits such as stability and control over one’s environment but also potential risks like financial strain. Therefore, while homeownership may not have provided significant discriminatory power in this study and did not predict the study outcome, it may play a critical role in other contexts.

The finding that certain variables differed significantly among the three income groups, but not total cholesterol and eGFR, is intriguing. This aligns with previous research suggesting that SES impacts various health outcomes differently. For instance, a study by Stringhini et al. [23] found that low SES was associated with a higher mortality risk from respiratory and digestive diseases but not from other causes. This suggests that SES’s impact on health outcomes can be disease-specific, which may explain why total cholesterol and eGFR were not significantly different among the income groups in this study.

The survival analysis results underscore the need to address socioeconomic disparities in CKD management and progression. This aligns with the broader literature on health disparities, consistently showing that lower SES is associated with worse health outcomes. For example, it is well demonstrated that individuals with lower SES have higher mortality rates across various diseases [24]. This highlights the critical need for healthcare providers to consider patients’ socioeconomic circumstances when developing treatment strategies.

This study’s methodology, particularly the use of bootstrap analysis for internal validation, adds robustness to the findings. However, taking into account the fact that 198 patients out of 759 were not included in this study, because of missing data about income, it is worth noting that this study’s context may limit the generalizability of the findings. The study population was drawn from Southern Italy, which may have unique socioeconomic structures and healthcare systems that differ from other regions or countries. Furthermore, we did not externally validate the composite SES score, and this undertaking remains a fundamental research step for the full validation of the same score. Finally, the observational design of this study does not allow definitive conclusions to be drawn in establishing causality between SES and health outcomes in CKD patients.

In conclusion, this study proposes a simple indicator of SES for CKD patients based on income and education. This indicator, which predicts clinical outcomes, further underscores the importance of considering socioeconomic circumstances in CKD patients. Future research should continue to explore this area, integrating various SES indicators to provide a more nuanced understanding of their role in CKD and inform more effective, equitable treatment strategies.

## Figures and Tables

**Table 1:** Baseline demographic, clinical characteristics, and quality of life questionnaire by declared income.

	No Declared Income (n = 112)	Declared Income from Retirement (n = 293)	Declared Income from Work or Work plus Retirement (n = 156)	*p*-Value
Age, year	57 ± 13	67 ± 7	53 ± 11	<0.001
Male gender, %	23%	66%	78%	<0.001
*Educational level, %*				
≤primary school	53%	62%	24%	<0.001
≥middle school	47%	38%	76%	
*Homeownership*, (%)	90%	90%	92%	0.660
*Average number of cars per capita*, %				
No car	13%	18%	3%	<0.001
Less than one	81%	70%	81%	
One or more	5%	12%	15%	
*Alcohol use,* %				
No	87%	63%	65%	<0.001
Current	13%	30%	27%	
Former	1%	7%	8%	
*Smoking*, %	27%	53%	58%	<0.001
*Diabetes*, %	27%	42%	20%	<0.001
*Hemoglobin*, g/dL	12.6 ± 2	12.9 ± 2	13.2 ± 2	0.027
*Total cholesterol*, mg/dL	192 ± 43	187 ± 45	187 ± 43	0.679
*Systolic blood pressure*, mmHg	132 ± 19	136 ± 17	129 ± 18	<0.001
*CRP high sensitivity*, mg/L	2.1 (0.9–5.9)	2.5 (1.1–5.4)	1.9 (0.9–4)	0.036
*Phosphate* mg/dL	3.9 ± 0.7	3.6 ± 0.8	3.7 ± 0.7	0.001
*eGFR*, mil/min/1.73 m^2^	33.4 (25.1–43.6)	35.8 (26.3–45.2)	35.8 (25.7–47.6)	0.321
*Mental component score*, MCS	42 (33.2–51.4)	45.6 (34–53.2)	46.5 (37.2–54.1)	0.041
*Physical component score*, PCS	45.1 (34.7–51.4)	42 (33.9–48.9)	46.2 (39.7–52.3)	<0.001

**Table 2:** Univariable and multivariable Cox regression analyses.

	Hazard Ratio, 95% CI, and *p* Value			
	Crude Model	Model 1	Model 2	Model 3
*Declared income source*				
Income from work or work and retirement	1 *	1 *	1 *	1 *
With income from retirement	3.85 (2.13–9.53).	2.07 (0.98–5.21)	1.96 (0.93–4.87)	2.01 (1.02–5.44)
	*p* = 0.002	*p* = 0.070	*p* = 0.078	*p* = 0.066
With no income	2.50 (1.18–9.53).	2.89 (1.18–7.79)	2.8 (1.16–8.3)	2.72 (1.16–7.72)
	*p* = 0.016	0.015	*p* = 0.019	*p* = 0.020
*Age*, year		1.07 (1.03–1.11)	1.07 (1.03–1.12)	1.07 (1.03–1.12)
		*p* = 0.001	*p* = 0.002	*p* = 0.001
*Male sex*		2.3 (1.2–4.66)	2.19 (1.14–4.45)	3.13 (1.51–6.53)
		*p* = 0.011	*p* = 0.020	*p* = 0.002
*From middle school education onward to lower or no education*		1.65 (1.08–2.67)	1.69 (1.12–2.81)	1.67 (1.04–2.95)
		*p* = 0.023	*p* = 0.018	*p* = 0.034
*Smoking*, yes		1.21 (0.7–2.22)	1.25 (0.7–2.41)	1.19 (0.68–2.43)
		*p* = 0.480	*p* = 0.483	*p* = 0.572
*Alcohol use*				
No		1 *	1 *	1 *
Current		0.57 (0.32–0.97)	0.59 (0.31–0.99)	0.55 (0.3–0.95)
		*p* = 0.041	*p* = 0.059	*p* = 0.027
Former		1.13 (0.51–2.1)	1.11 (0.48–2.2)	1.03 (0.41–2.15)
		*p* = 0.743	*p* = 0.764	*p* = 0.929
*Diabetes*, yes			1.32 (0.83–2.09)	1.1 (0.68–1.81)
			*p* = 0.227	*p* = 0.690
*Hemoglobin*, g/dL				0.84 (0.73–0.98)
				*p* = 0.023
*Systolic blood pressure*, mmHg				1 (0.99–1.01)
				*p* = 0.807
*Phosphate* mg/dL				1.34 (0.99–1.84)
				*p* = 0.050

* Reference category.

**Table 3:** SES composite risk score.

	Risk Score (%)
High income and high educational level	0
High income and low educational level	23
Intermediate income and high educational level	32
Low income and high educational level	45
Intermediate income and low educational level	55
Low income and low educational level	68

## Data Availability

The data presented in this study are available on request from the corresponding author. The data are not publicly available due to privacy.

## Supplementary Materials

The following supporting information can be downloaded at: https://www.mdpi.com/article/10.3390/jcm13123600/s1, Table S1. Baseline characteristics of included and excluded patients. Table S2. Patient interviews.
