# Supplementary material for: Socioeconomic Status and Clinical Outcomes in Chronic Kidney Disease: Bootstrap Validation of a Simple Indicator
Source: J Clin Med. 2024 Jun 20;13(12):3600. doi: 10.3390/jcm13123600 (PMC11204512; doi:10.3390/jcm13123600)
Supplement: Supplementary file 1 [file jcm-13-03600-s001.zip › jcm-2992654-supplementary.pdf]

Supplementary Table S1. Baseline characteristics of included and excluded patients

	Excluded (n=198)	Included (n=561)	p-value
Age, year	63± 9	61± 11	0.343
Male gender, %	58%	61%	0.533
Smoking, %	51%	49%	0.661
Alcohol use, %			
no	69%	68%	0.908
current	26%	26%	
former	5%	6%	
Diabetes, %	41%	33%	0.040
Haemoglobin, g/dL	12.5± 1,8	12.9± 1,8	0.002
Total cholesterol, mg/dL	183± 46	188± 44	0,226
Systolic blood pressure, mmHg	136± 18	133± 18	0.014
CRP high sensitivity, mg/L	2.7 (1.3-6.8)	2.2 (1-5)	0.009
Phosphate mg/dL	3.8± 0,8	3.7± 0.7	0.635
eGFR, mil/min/1.73 m^2	35± 14	36± 13	0.191

Data are mean and standard deviation, median and interquartile range, or absolute number and percentage, as appropriate.

Supplementary Table S2

PATIENT'S ID ___________

Date____/____/___

Do you carry out work activities?

☐ **YES**

☐ **NO**

Are you retired from work?

☐ **YES**

☐ **NO**

Please indicate the number of cohabitants_________________

Please indicate the number of cars of your family/cohabitants____________

Please indicate the highest educational level

☐ Illiterate
☐ Elementary school
☐ Middle school
☐ High school
☐ University degree
